# Turning induced plasticity into refined adaptations during range expansion

**DOI:** 10.1038/s41467-020-16938-7

**Published:** 2020-06-26

**Authors:** Ahva L. Potticary, Erin S. Morrison, Alexander V. Badyaev

**Affiliations:** 1 Department of Ecology & Evolutionary Biology, University of Arizona, Tucson, AZ 85721 USA; 2 Liberal Studies, New York University, New York, NY 10003 USA

**Keywords:** Morphogenesis, Evolutionary ecology, Evolutionary developmental biology

## Abstract

Robustness against environmental fluctuations within an adaptive state should preclude exploration of new adaptive states when the environment changes. Here, we study transitions between adaptive associations of feather structure and carotenoid uptake to understand how robustness and evolvability can be reconciled. We show that feather modifications induced by unfamiliar carotenoids during a range expansion are repeatedly converted into precise coadaptations of feather development and carotenoid accommodation as populations persist in a region. We find that this conversion is underlain by a uniform and coordinated increase in the sensitivity of feather development to local carotenoid uptake, indicative of cooption and modification of the homeostatic mechanism that buffers feather growth in the evolution of new adaptations. Stress-buffering mechanisms are well placed to alternate between robustness and evolvability and we suggest that this is particularly evident in adaptations that require close integration between widely fluctuating external inputs and intricate internal structures.

## Abstract (web summary)

Phenotypic robustness to environmental variation is seemingly at odds with evolvability. Here, the authors analyze carotenoid use and accommodation in feather development across a recent avian range expansion and show that cooption of a stress-buffering mechanism can reconcile robustness and evolvability.

## Introduction

There is an evolutionary tension between the preservation of functioning phenotypes and the exploration of alternative phenotypic states^1–3^. Cooption of stress-buffering mechanisms that maintain each adaptive state^4–6^ is thought to bridge sequential adaptive states^7–12^. However, it is difficult to infer the historical role of stress-buffering mechanisms from comparisons of contemporary adaptations alone, because homeostatic systems necessarily suppress variation that interferes with current functioning and new adaptations subsume preceding adaptations in evolution^13^. Thus, extant adaptations are not each other’s evolutionary stages, even in related species, and the debate on whether transitions between adaptations are more concordant with functionally relevant (i.e., adaptive) or stress-induced (i.e., nonadaptive) contemporary variation is often inconclusive^14–19^. A more inferential approach is to study transitions between adaptations as a process. This requires an empirical system where it is possible to observe both the developmental effects of an environmental factor that has varied during the evolution of the lineage and changes in its developmental effects over time. Here, we show that an ongoing range expansion of a passerine bird satisfies these criteria by enabling direct observation of sequential historical changes in how growing feathers accommodate the diverse carotenoids that color them.

Many bird species color their feathers with carotenoids acquired from the diet. Carotenoid metabolic networks are well-characterized for many bird species, allowing classification of feather carotenoids into dietary carotenoids (those obtained directly from the diet and deposited into feathers unchanged) and metabolized carotenoids (those biochemically converted by birds prior to deposition in feathers)^20^. Further, some metabolized carotenoids are interchangeably derived from several dietary carotenoids (e.g., Supplementary Fig. 1)—a phenomenon known as metabolic degeneracy^21^. The more degenerate a metabolized carotenoid compound is, the more reliably it will be produced across a range of environments by conversion of variable dietary carotenoids. Metabolized carotenoids, especially the degenerate compounds, are thus more reliably present across environments and have likely had a longer time to coevolve with and integrate into other organismal systems than dietary carotenoids or their nondegenerate metabolized derivatives^22,23^.

**Fig. 1:**
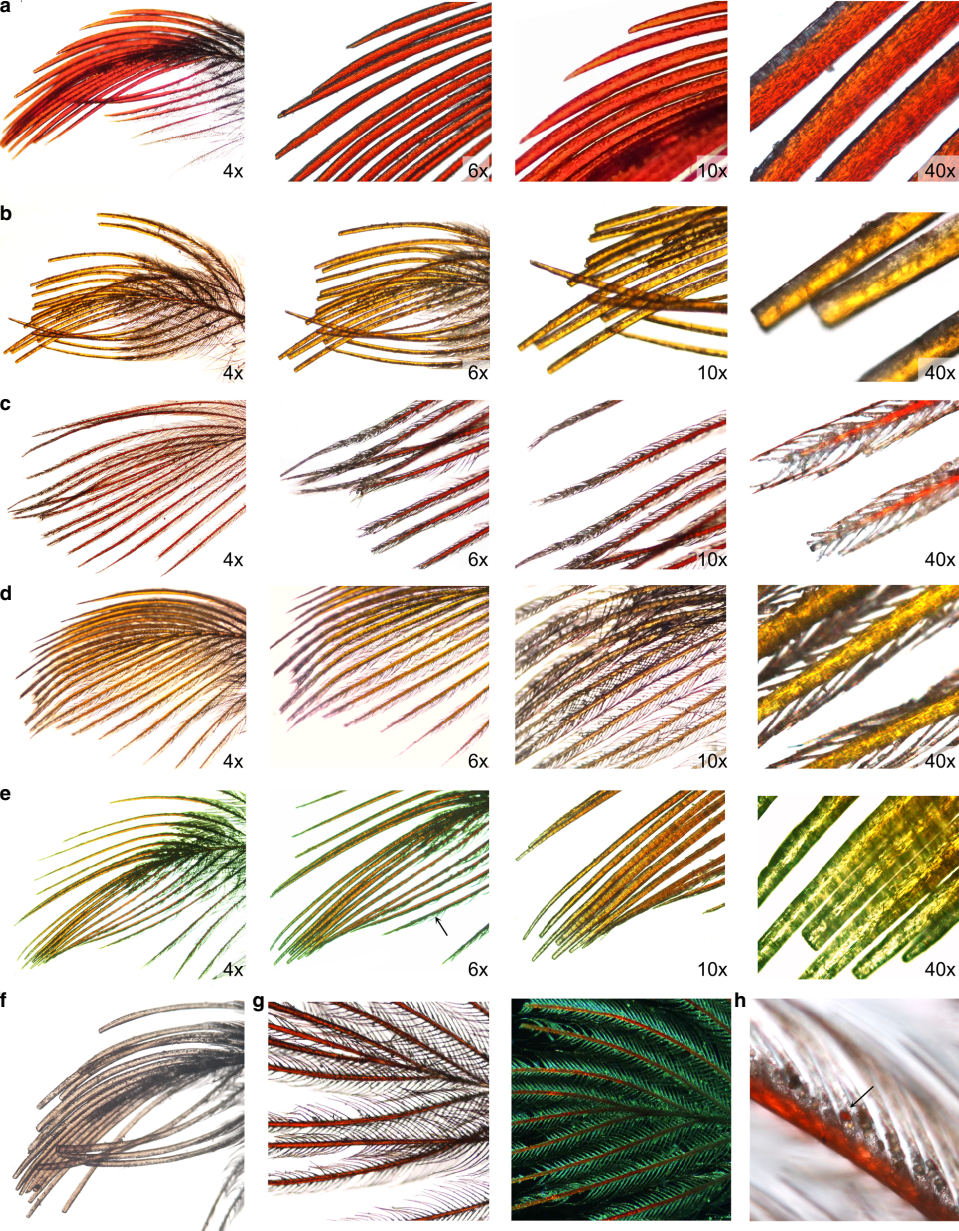
Feather differentiation responses to carotenoid uptake in house finches. **a**, **b** the full response—complete lack of barbule differentiation, **c**, **d** no response—complete differentiation, **e** the partial response—partial differentiation with either few differentiated barbs or parts of individual barbs differentiated (6× panel, arrow), **f** an example of mismatch of anticipatory response in feather differentiation—i.e., lack of differentiation in the absence of carotenoid uptake, **g** typically, in fully-differentiated feathers, only the rami of barbs contain carotenoids, but **h** unusually thick barbules occasionally trap carotenoids (arrow). Only crown feathers are shown to facilitate comparison.

**Fig. 2:**
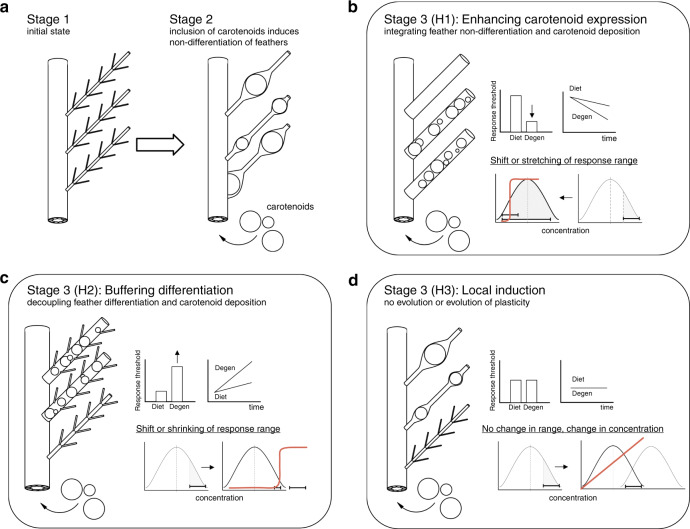
Proposed pathways for feather differentiation and carotenoid uptake coevolution. **a** Stage 1 (default state of fully-differentiated barbules) and Stage 2 (induction of barbule non-differentiation by novel carotenoid uptake) are well-documented. Subsequent Stage 3 depends on the mechanisms underlying the feather-carotenoid association. We test three alternative scenarios (predicted responses shown in red). **b** *H1*: selection for enhancing carotenoid expression strengthens the link between feather non-differentiation and carotenoid uptake, predicting a lower threshold (higher sensitivity) of response (shown as a bracket) to carotenoid concentration (gray area under concentration curve), especially for degenerate carotenoids and in older populations. **c** *H2:* selection for restoring feather differentiation and buffering it from disruption by novel carotenoid uptake, predicts a higher threshold of response (lower sensitivity) to carotenoid concentration, especially for degenerate carotenoids and in older populations. **d** *H3:* no evolutionary change—i.e., continuation of Stage 2 where feather plasticity allows feather differentiation in proportion to local concentration of carotenoids (shifting concentration curves and proportional feather response). No difference in response to degenerate and dietary carotenoid accumulation is predicted. Supplementary Fig. 3 outlines tests of these hypotheses.

The incorporation of carotenoids into the barbs of growing feathers commonly causes feather structural modifications that range from transient malformations to intricate co-evolutionary adjustments^24–26^. A particularly striking modification is the effect of carotenoid uptake on an iconic feature of feathers—interlocking barbules (Supplementary Fig. 2)—which are responsible for a variety of functions, from water repellency and insulation to flight. For nearly a century, it has been known that the uptake of carotenoids into barbs often interferes with barbule development (hereafter “feather differentiation”; Fig. 1a, c), underpinning a textbook explanation for why feathers that are structurally specialized, such as flight and down feathers, are buffered against significant carotenoid uptake and are rarely colorful^27^. Yet, the relationship between feather differentiation and carotenoid input is highly variable: in some species, carotenoid-bearing feathers develop barbules that subsequently break off to enhance ornament display, in others, feathers develop deformed or partial barbules, while yet in others, feathers completely lack barbules from the onset of development, which decreases diffuse scattering and amplifies color intensity^25,26,28^. The extent to which phenotypic associations such as these are stages of an evolutionary process has not been studied. Thus, it is not known whether the feather modifications resulting from uptake of external carotenoids are adaptations to maximize coloration (Fig. 2b), transient malformations that require subsequent evolution of greater buffering of feather growth (Fig. 2c), or evidence of evolved plasticity, where local carotenoids directly induce feather growth modification^29,30^ (Fig. 2d).

**Fig. 3:**
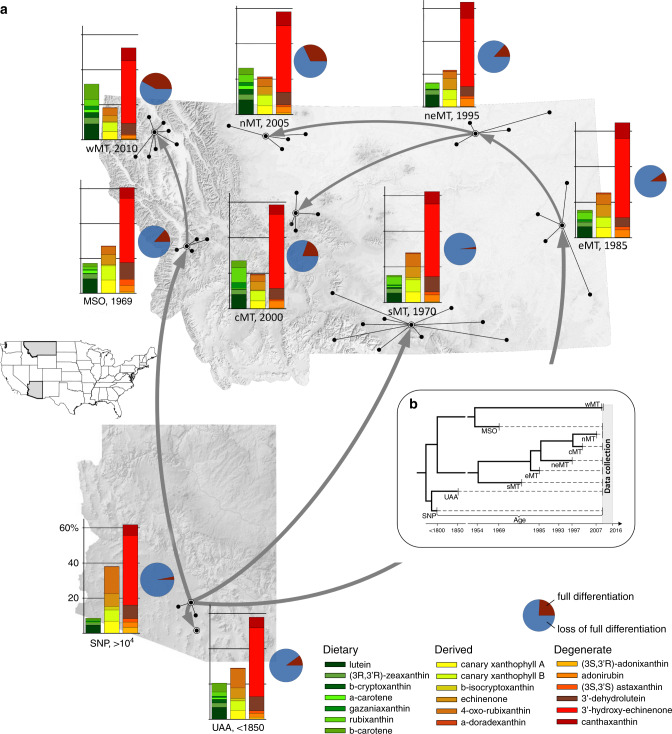
Historical changes in feather differentiation in response to carotenoid uptake. **a** Shown are regional summaries (Supplementary Table 1) for individual populations (dots connected by lines) and the year of population establishment in Arizona and Montana (shaded on United States map). Gray arrows link sequentially established populations. Feather differentiation is % of individuals showing response (Supplementary Table 2), carotenoid uptake is proportion of a given carotenoid in a feather sample, averaged across ornaments (Supplementary Data 1, 2). Birds in older populations accumulated a greater proportion of metabolically-derived carotenoids than dietary carotenoids. **b** Known time and sequence of population establishment allows historical staging for testing predictions in Fig. 2. Shown are populations, years of their establishment, and population age at the time of data collection (dashed vertical area).

Here, we test these scenarios using the recent colonization of western North America by house finches (*Haemorhous mexicanus*), which has produced replicated, hierarchical sequences of known-age populations across one of the widest ecological ranges of any extant bird species^31^(Fig. 3). From their native range in southwestern North America to their newly established range, house finches are exposed to diverse plant communities that provide widely distinct sources of carotenoid precurors^32^, use different subsets of a carotenoid-producing metabolic network, and show an array of feather modifications in response to carotenoids^22,33^. These modifications, illustrated in Fig. 1, vary from a near complete loss of barbules in ancestral and older populations to full barbule development in new populations, with a variety of intermediate stages.

Here we examine whether this diversity represents evolutionary stages by tracing historical changes in developmental responses of feathers to the uptake of 19 carotenoids of varying biochemical derivation, along trajectories that connect three native populations in southern Arizona, and 40 recently established populations across Montana (Fig. 3, Supplementary Table 1). We contrasted feather differentiation responses to the accumulation of metabolically-degenerate carotenoids (Supplementary Fig. 1), which are nearly ubiquitous in house finch feathers across all environments, with feather responses caused by dietary carotenoids, whose occurrence and relative abundance in feathers varies across house finch populations (Supplementary Data 1, 2).

First, we test whether historical changes in the association between presence of feather barbules and carotenoid uptake are concordant with those predicted by evolution for greater display of carotenoid-based ornamentation, evolution of buffering mechanisms that decouple feather differentiation and carotenoid uptake, or mechanical interference, where plasticity of feather growth accommodates local carotenoids without long-term evolutionary consequences (Fig. 2). Because the mechanisms underlying these hypotheses depend on the familiarity of an evolving lineage with carotenoids, and because degenerate carotenoids have greater predictability and integration with organismal processes that dietary compounds, these hypotheses make opposite predictions for the effects of degenerate vs. dietary carotenoids in the transitions between feather-carotenoid associations (Fig. 2b–d). To test these hypotheses, we investigated the historical changes in both the carotenoid concentration that induces feather structure response and the shape of the response across populations (Supplementary Fig. 3).

**Fig. 4:**
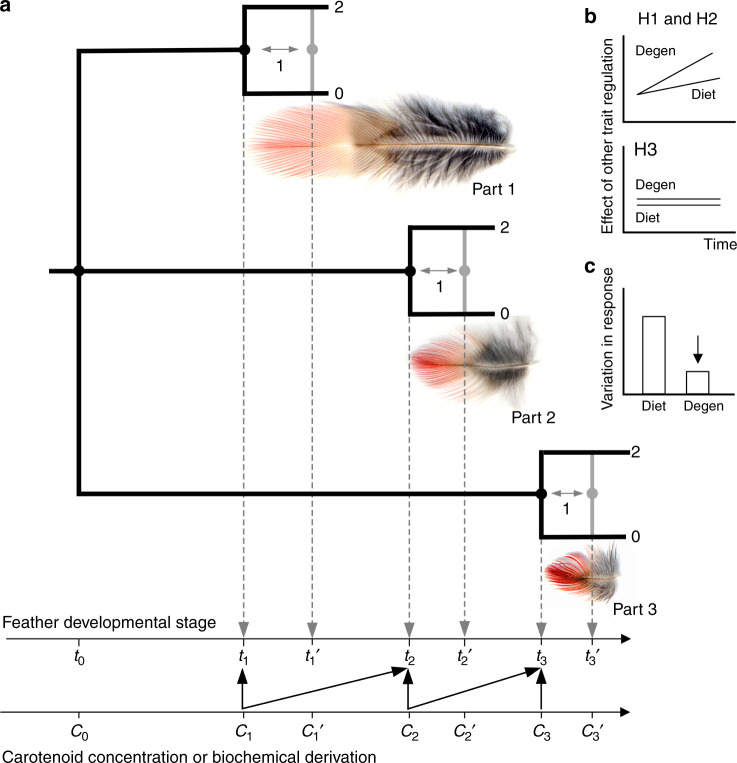
Evolution of regulation of feather differentiation. **a** The tip is the oldest part of a feather and a presumed time of developmental decision (*t*_n_) that determines whether barbules separate from barbs, ultimately producing either a non-differentiated (2) or differentiated (0) feather in response to carotenoid uptake (Supplementary Fig. 2). Partial differentiation (1) can be produced by shifting the decision point to later developing, more basal, parts of the ornamental feather (*t*_n_′). Within-individual heterochrony of the molt of ornamental areas (rump: Part 1, breast: Part 2, and crown: Part 3 are shown as examples) allows assessment of organismal coordination of feather differentiation decisions. **b** H1 and H2 (Fig. 2) predict a greater anticipatory response of feather differentiation to carotenoids, affecting differentiation of earlier molting parts (e.g., angled arrow *c*_1_ → *t*_2_, between carotenoid concentration at the time of the differentiation decision of Part 1 feathers and that of Part 2 feathers), especially for degenerate carotenoids and in older populations. H3 predicts no such anticipatory response, but consistent induction of feather differentiation by carotenoids available at the time of feather growth (e.g., vertical arrow *c*_2_ → *t*_2_). **c** Greater organismal integration of feather differentiation decisions and a lower threshold response (Fig. 2b) should lead to greater precision (lesser variation) in response of feather differentiation to carotenoid uptake, especially in degenerate carotenoids (Supplementary Figs. 1b and 3).

Second, we capitalize on within-individual differences in the timing of molt of different ornamental areas (Fig. 4) to establish whether variation in feather differentiation is triggered locally by each feather’s own carotenoid uptake, or reflects an organism-wide response where carotenoid accumulation in earlier molting feathers influences the development of later growing feathers (i.e., anticipatory response). We then examine historical changes across populations in the coordination of feather responses across ornaments and discuss potential regulatory mechanisms behind the three main hypotheses (Fig. 4).

Across an array of diverse carotenoids, and thus a diversity of starting points, we uncover a remarkably uniform historical increase in the sensitivity of developing feathers to carotenoid uptake as populations persist in their environments. Within each population, the sensitivity of the developing feather to carotenoid uptake primarily depends on carotenoid metabolism (dietary, metabolized, and degenerate) and on a population’s familiarity with a particular suite of local carotenoids. These findings suggest that the coevolution of feather differentiation and pigment deposition coopts general mechanisms that buffer feather growth, where the initial disruption of feather differentiation by unfamiliar compounds is transformed into increasingly robust and diverse states of feather-carotenoid associations across populations (scenario of Fig. 2b, Supplementary Fig. 3c, d). Further, these results establish that the loss of barbules in carotenoid-bearing feathers are adaptations and explain the diverse associations between feather structure and carotenoid uptake across the ecological range of this species (Fig. 1). More generally, modification of the mechanisms that maintain robustness of each adaptive state facilitates transitions between subsequent adaptive states, illustrating an empirical reconciliation of robustness and evolvability.

## Results

### Carotenoids differ in variability and predictability

We measured structural differentiation in response to the accumulation of 19 carotenoids (Supplementary Fig. 1, Supplementary Data 1 and 2, Supplementary Table 2) in 3338 feathers from 1196 adult males from 43 populations (Supplementary Table 1). Across all populations, birds accumulated less variable concentrations of degenerate carotenoids and highly variable concentrations of dietary carotenoids (among-population coefficient of variation (CV) for all dietary carotenoids combined = 91.97% and for degenerate carotenoids combined = 58.7%, *F* = 224.82, *P* < 0.001; see Supplementary Data 1 and 2 for individual compounds). For example, only 3% of birds in the ancestral SNP (Arizona) population accumulated dietary β-cryptoxanthin in their feathers, compared with 50% in the youngest wMT (Montana) population, where birds also retained up to ten times higher concentration of this dietary compound (Supplementary Data 1). Similarly, SNP birds accumulated ten times more dietary α-carotene compared with neMT birds. Rubixanthin, whose main source is wild rose fruits and petals, occurred in all individuals and had up to six times higher concentration in feathers from the cMT and eMT populations compared with birds from the ancestral SNP population, where it only occurred in half of individuals (Supplementary Data 1). Even recently diverged and geographically adjacent populations differed in the accumulation of dietary carotenoids when occupying distinct environments: for example, neMT (prairie) and nMT (forested foothills of Continental Divide) populations showed fourfold differences in the retention of dietary β-carotene in their feathers (Supplementary Data 1).

### Familiar carotenoids exert stronger feather responses

**Fig. 5:**
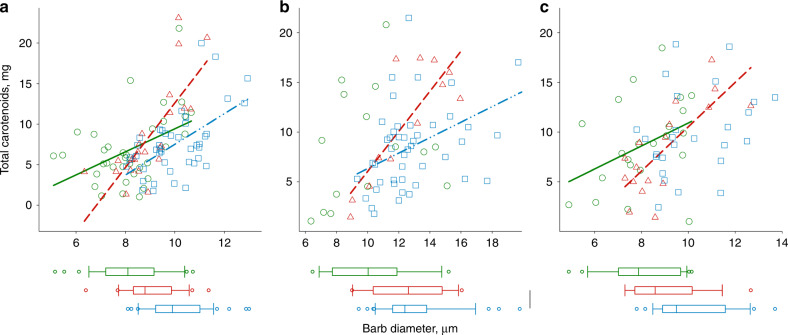
Undifferentiated feathers have thicker barbs and accumulate more carotenoids. Regressions of barb diameter on carotenoid amount in fully differentiated (“0”, green circles and green solid lines), partially-differentiated (“1”, red triangles and red dashed lines), and undifferentiated (“2”, blue circles and blue dash-and-dot lines) feathers as well as box plots of barb diameter (box shows medians with 25% ranges, line is the range between minimum and maximum values, dots are the outliers) for (**a**) breast (“0”: mean ± s.e.m 8.38 ± 0.27; standardized regression coefficient *b*_ST_ = 0.45, *t* = 2.93, *P* = 0.002; “1”: 8.98 ± 0.3, *b*_ST_ = 0.77, *t* = 5.60, *P* < 0.001; “2”: 9.27 ± 0.17_,_ *b*_ST_ = 0.57_,_ *t* = 4.50, *P* < 0.001; slope for “1” differs from others: MANOVA (Duncan test) *F* = 5.04, *P* = 0.01), (**b**) crown (“0*”:* 10.30 ± 0.7, *b*_ST_ = 0.25, *t* = 0.75, NS; “1”: 12.41 ± 0.7, *b*_ST_ = 0.82, *t* = 4.60, *P* < 0.01; “2”: 12.14 ± 0.37, *b*_ST_ = 0.44, *t* = 3.28, *P* = 0.02; slope for “1” differs from others: *F* = 2.96, *P* = 0.04), and (**c**) rump (“0”: 8.07 ± 0.33, *b*_ST_ = 0.39, *t* = 1.98, *P* = 0.06; “1”: 8.49 ± 0.38, *b*_ST_ = 0.77, *t* = 4.65, *P* < 0.01; “2”: 8.86 ± 0.24, *b*_ST_ = 0.33, *t* = 1.55, NS; slopes do not differ: *F* = 2.86, *P* = 0.06) feathers. In all ornamental areas, feather differentiation categories had different barb diameters (Supplementary Table 2, Student–Newman–Keuls tests, *P* < 0.05), except for partially and fully-differentiated groups in (**b**). Based on Supplementary Data 1 and 2 and Supplementary Table 2.

In the ancestral population, only 4% of birds showed full differentiation of barbules (Fig. 3a). However, within a few generations of colonizing new environments and accumulating different combinations of carotenoids (Supplementary Data 1, 2), finches showed full or partial feather differentiation (Fig. 3). Feathers that did not develop barbules had wider barb diameters than differentiated feathers, which was associated with greater accumulation of carotenoids (Fig. 5).

**Fig. 6:**
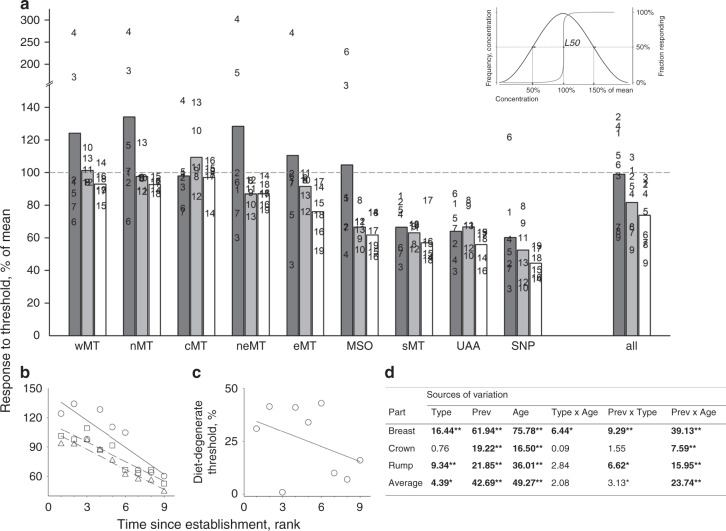
Familiar carotenoids exert stronger feather response. **a** Response threshold (% of mean concentration that exerts a full response in 50% of individuals in a population, *L50*, inset) for dietary (dark gray), derived (gray), and degenerate (white) carotenoids in feathers across populations arranged by the time since establishment, from the youngest (wMT) to the oldest (SNP). Response curve in inset shows *L50* coinciding with a mean concentration (100% of mean). When a higher than average concentration is required to exert full response in 50% individuals, the response curve shifts to the right (values > 100% mean, above dashed line), when less than average, the curve shifts to the left (values < 100% mean, below dashed line). Numbers in population graphs correspond to dietary carotenoids: 1—lutein; 2—(3R,3′R)-zeaxanthin; 3—β-cryptoxanthin; 4—α-carotene; 5—gazaniaxanthin; 6—rubixanthin; 7—β-carotene; derived nondegenerate carotenoids: 8—canary xanthophyll A; 9—canary xanthophyll B; 10—β-isocryptoxanthin; 11—echinenone; 12—4-oxo-rubixanthin; 13—a-doradexanthin; and degenerate carotenoids: 14—(3S,3′R)-adonixanthin; 15—adonirubin; 16—(3S,3′S) astaxanthin; 17—3′-dehydrolutein; 18—3′-hydroxy-echinenone; 19—canthaxanthin. Numbers in the combined (“all”) graph are population age ranks from young to old: 1—wMT, 2—nMT, 3—cMT, 4—neMT, 5—eMT, 6—MSO, 7—sMT, 8—UAA, 9—SNP. Graph shows average responses across feathers in three ornamented areas. **b** Older populations have lower response threshold to dietary (circles, solid line), derived (squares, dashed line), and degenerate carotenoids (triangles, dash-dot line), and **c** more similar responses to dietary and degenerate carotenoids. **d** Effect of a carotenoid metabolic derivation (*Type*: diet, derived, degenerate) and prevalence in feathers (Prev, Supplementary Data 1 and 2), as well as a population’s age rank (Age), and their interactions on differentiation response in breast, crown, and rump feathers (Supplementary Data 4), and averaged within individual. Older populations, more common and degenerate carotenoids have lower response thresholds, although the importance of carotenoid’s prevalence in a population depends on population’s age. Bold values are significant at *P < 0.05 and at **P < 0.01 after within-ANCOVA model Bonferroni adjustment.

For each carotenoid compound in each population, we estimated the relative concentration needed to produce feathers without barbules in 50% of individuals (L50), and also established whether the response curve was linear or threshold-like (Methods, Supplementary Fig. 3, Supplementary Data 4). We found that, in all populations, degenerate carotenoids caused barbule loss at much lower concentrations than did dietary carotenoids, with metabolized nondegenerate carotenoids being intermediate (Fig. 6a). Across all carotenoids, the response threshold uniformly decreased with population age and greater prevalence of local carotenoids in feathers (frequency of occurrence among individuals; Fig. 6b, d; Supplementary Data 1). Further, the difference in response to dietary and degenerate carotenoids decreased with population age (Fig. 6c).

### Feather responses differ mostly in sensitivity

**Fig. 7:**
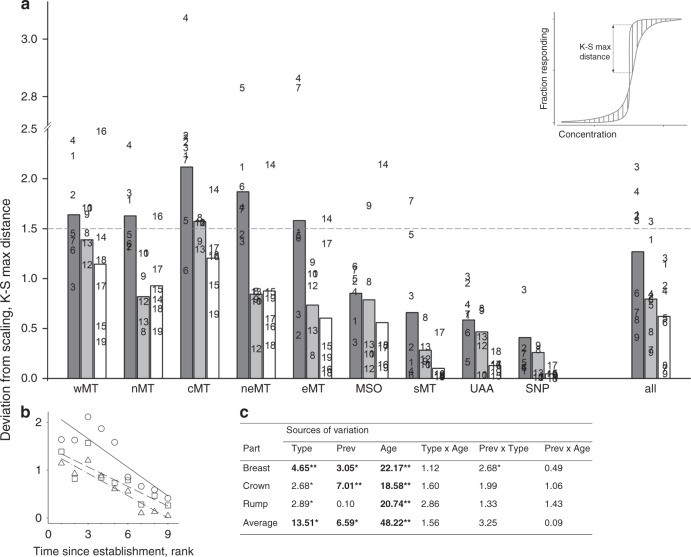
Scaling of feather responses to carotenoid uptake. **a** Residuals (absolute values) of an AFT regression (that removes differences due only to scaling of response) of dietary (dark gray), derived (gray), and degenerate (white) carotenoid concentrations on the likelihood of feather response across populations arranged by the time since establishment, from the youngest to the oldest. Inset: Kolmogorov–Smirnov (K–S) maximum distance (double-headed arrow) between estimated and observed scaling functions assesses the probability of observing this distance (vertical lines) by chance; |K–S max distances| <1.49 are not different from 0 under *P* < 0.05 (Supplementary Fig. 4, Methods). Responses under dashed line come from the same underlying distributions and differ by the sensitivity of response only. Differences in response above the dashed line are not explained by simple rescaling of carotenoid concentration (Supplementary Data 4). **b** Feather responses to carotenoid uptake show less variation and smaller deviation from scaling with increasing population age. **c** Feather response to degenerate carotenoids and derived carotenoids in all but one population (cMT), differs only in sensitivity, whereas response to accumulated dietary carotenoids shifts to a predominantly linear response in young populations, and to threshold-like in older populations. Based on Supplementary Data 4, symbols, numbers and abbreviations as in Fig. 6a, d. Bold values are significant at *P < 0.05 and at **P < 0.01 after within-ANCOVA model Bonferroni adjustment.

We then asked whether the observed historical changes in thresholds of carotenoid concentration that causes feather responses (Fig. 6a) were specific to carotenoid type or resulted from changes in sensitivity of response (i.e., scaling of response curve, Supplementary Fig. 3c). We fitted an accelerated failure time (AFT) regression to all feather responses within ornamental areas (populations combined, see Methods) to remove the effect of scaling, such that population- or carotenoid-specific responses that only differed in sensitivity to a given concentration (Supplementary Fig. 3c), would have an identical distribution of residuals from this regression. We found that, with the exception of adonixanthin (which had linearly-proportional effects on feather response in four populations, Supplementary Data 4, Supplementary Fig. 3d), the responses to metabolically-derived carotenoids across all populations and ornaments differed only in sensitivity (i.e., a decrease or increase in population response in relation to a given carotenoid concentration, Supplementary Fig. 3c). Thus, across most metabolized carotenoids, the observed decrease in response threshold with population age (Fig. 6a, b, d) was caused by increased sensitivity (Fig. 7a, b; Supplementary Data 4). In contrast, for dietary carotenoids, we observed a shift from primarily linearly-proportional responses [especially to carotenoids infrequently found in feathers (Fig. 7c, Supplementary Data 4)] in recently established populations, to a threshold-like response in older populations (Supplementary Data 4), where the sensitivity of response progressively increased with populations age (Fig. 7a, c; Supplementary Fig. 3d).

### Organismal coordination of feather responses to carotenoids

**Fig. 8:**
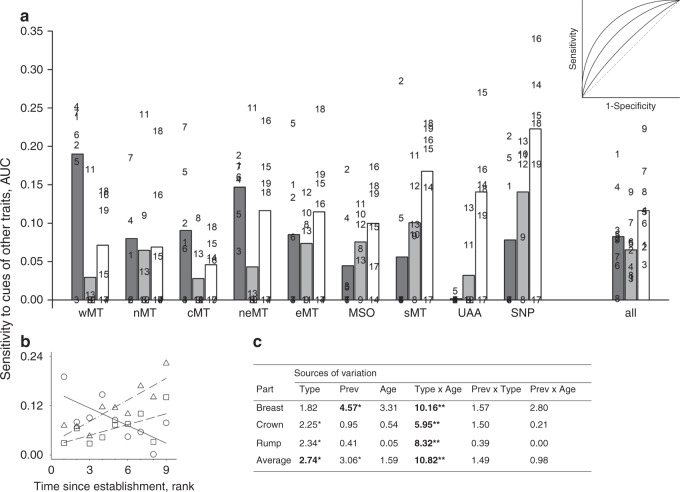
Organismal coordination of feather response. **a** Sensitivity of feather differentiation in a local ornamental area to carotenoid accumulation causes differentiation in a different ornamental area (Fig. 4a). Shown are areas under curve (AUC) above the diagonal of random response (inset) for dietary (dark gray), derived (gray) and degenerate (white) feather carotenoids across populations arranged by the time since establishment, from the youngest to the oldest. Inset: in a logistic regression, the number of individuals predicted to show a response to a given cue divided by the number of individuals observed to have a response is the sensitivity, and the number of individuals predicted to have no response to the same cue divided by the number of individuals observed not to have a response is the specificity. The diagonal of the plot of (1-specificity) by sensitivity corresponds to an equal probability of true positives and false negatives (i.e., random choice). Values above the diagonal, measured as AUC, indicate significant sensitivity of response (see Methods for tests). **b**, **c** Decreasing organism-wide coordination of responses to dietary carotenoids in feathers is replaced with increasing coordination of responses to derived and degenerate carotenoids as populations age. Based on Supplementary Data 4, symbols, numbers and abbreviations as in Figs. 6a and 7c. Bold values are significant at *P < 0.05 and at **P < 0.01 after within-ANCOVA model Bonferroni adjustment.

Having established that the feather response to carotenoid input is determined by consistent increase in sensitivity of feather development to local carotenoid uptake (Fig. 7), we investigated the regulation of this precision. We capitalized on the temporal ordering of developmental events within a feather (Supplementary Fig. 2), and the different timing of feather growth across ornamental patches within an individual (Fig. 4), to determine whether feather responses were anticipatory (a coordinated uniform response across feathers despite differences in local carotenoid uptake) or locally induced (each ornaments’ feathers are independently modified by local uptake of the carotenoids circulating at the time of its’ development). We adapted a statistical technique that allowed us to directly compare feather responses from linear, accelerating, and threshold distributions (Methods). We found that young populations showed greater coordination of barbule loss across ornaments in response to dietary carotenoids (Fig. 8), especially those most frequently found in feathers (e.g., zeaxanthin, lutein, β-carotene, Supplementary Data 4). These anticipatory responses might have enabled birds in these populations to accumulate greater amounts of dietary carotenoids (Fig. 3a). As populations persisted in their environment, anticipatory responses to dietary carotenoids notably diminished, and were replaced with anticipatory responses to derived carotenoids (Fig. 8, Supplementary Data 2). Interestingly, these anticipatory responses occasionally resulted in mismatches between feather differentiation and actual carotenoid uptake. For example, even when they did not receive pigment themselves, some later molting feathers did not develop barbules when earlier molting ornamental feathers accumulated carotenoids (Fig. 1f).

## Discussion

We traced historical trajectories of organismal accommodation of novel external inputs, from induction and proliferation of stress-like effects in young populations, to progressive buffering and fine-tuning of local responses as inputs become familiar in older populations. We observed that, following introduction into a novel environment, house finches express full structural differentiation in ornamental feathers (a response which had been largely lost in older populations), and then gradually lose it again, but in response to different combinations of carotenoids. This sequence is congruent with the scenario outlined in Fig. 2b, establishing a lack of barbule development in carotenoid-bearing feathers as an adaptation, which evolved by strengthening the link between barbule loss and carotenoid uptake. We further document a remarkably uniform increase in feather response sensitivity across an array of biochemically distinct carotenoids (Fig. 7a), and show that this increase is underlain by familiarity with a carotenoid—reflected by both its prevalence within population and its metabolic degeneracy, which corresponds to a longer coevolution time (Figs. 6, 7). Lastly, both induced responses in new populations and the maintenance of specific responses in old populations were accomplished through organism-wide coordination of responses (Fig. 8a).

Taken together, these findings suggest that the association between feather differentiation and carotenoid accumulation evolved though cooption and modification of the mechanisms that normally shield feather growth from incorporation of unwanted components from plasma or regular pigment uptake. Two aspects of these mechanisms are particularly relevant to our results—the link between total concentration of accumulated carotenoids and feather growth differentiation^34^ and evolving selectivity of feather follicular membrane for chaperoning carotenoids into developing feather^35–38^.

The elements of feather growth most closely associated with carotenoid uptake are the barb addition rate and initial diameter of the carotenoid-bearing ramus of the barb (Supplementary Fig. 2). Both of these elements are determined prior to a feather’s elongation^34^, such that feather differentiation is predisposed to be affected by prior carotenoid presence in feather follicle and the mechanisms of feather differentiation can either capitalize on it or buffer against it. The lack of carotenoid specificity in feather differentiation, as well as the organism-wide coordination of feather responses found in this study, suggest shared regulation of carotenoid processing, delivery, feather growth and differentiation. A particularly likely candidate for this regulation is modification of the mechanisms that determine the structure of non-ornamental feathers across the avian body. Barbule separation is routinely and reversibly truncated in many feather types in the absence of any pigment uptake^39,40^. Similar patterns of feather modification may be produced by biochemically distinct carotenoids if the mechanisms mediating carotenoid uptake can coopt the developmental processes that influence barbule development. Several lines of evidence support this hypothesis and point to integration of carotenoid deposition with general mechanisms of feather growth and differentiation.

First, barbule separation is the earliest feather differentiation decision at the level of the barb^41,42^ (Supplementary Fig. 2, Fig. 4), preceding the formation of the rami, where carotenoids are deposited (Supplementary Fig. 2b). We found that barb diameters were wider on undifferentiated feathers (Figs. 1, 5), suggesting that accumulating barbule cells develop and fuse to the rami of the barb, rather than barbule development being truncated overall. Such early and irreversible non-differentiation can be triggered by either threshold-like effects of carotenoid accumulation in the focal follicle, or by anticipatory, organism-wide coordination based on carotenoids circulating in the plasma (Fig. 4b).

Second, the finding of partial differentiation of feathers in new populations (Fig. 1e) suggests involvement of the mechanisms underlining the hierarchical organization of feather development^42–44^. For example, carotenoids may truncate the development of barbules overall, or initiate keratinization of the barb prior to the differentiation of barbules. The length of a single-cell-wide individual barbule depends on the height of the barb ridge (Supplementary Fig. 2), which is determined by the rate of cell differentiation and growth in the ramogenic zone^42^. Intriguingly, production of growth-inducing Sonic Hedgehog (Shh) in the marginal plate (Supplementary Fig. 2) occurs while Shh receptors are present only in the barbule, not the marginal plates^45^, indicating that the marginal plate may be the signaling center that regulates barbule cell proliferation and differentiation. If presence of carotenoids in feather follicles affect growth of the marginal plate cells^45^, as shown in some species^46,47^, then carotenoid uptake will directly influence barbule segregation. While experimental confirmation is needed, the common denominator of all these scenarios is that carotenoid uptake alters general, not pigment specific, mechanisms that normally maintain, and regulate feather growth.

Consumed and metabolized carotenoids differ in the rate and location of their absorption in the gut^48–50^, the type of plasma lipoproteins they are transported by^51,52^, and their ability to diffuse into a feather follicle^53–55^ and bind to keratin of the feather matrix^56,57^. Given this diversity, what mechanisms could produce the observed organism-wide coordination of feather responses (Fig. 8)? And what accounts for the scaling of feather developmental responses to distinct carotenoids across historical contexts (Fig. 7)? Our findings suggest that the mechanisms that buffer growing feathers and maintain population robustness of carotenoid-feather associations may also channel transitions between them. A particularly good candidate that may link sensitivity to plasma circulating carotenoids with the developmental and evolutionary aspects of feather growth is the evolution of follicular membrane permeability. Carotenoids penetrate feather follicles attached to plasma lipoproteins and follicular membranes are thought to mediate the incorporation of carotenoids into developing barbs (Supplementary Fig. 2). Interestingly, uncommon carotenoids are known to overwhelm follicular membrane buffering, resulting in failure of the membrane as a selective barrier and passive shunting of novel carotenoids into growing feathers, often with cascading effects on feather structure^35–37^. Evolving selectivity in membrane permeability may explain why historical familiarity with a particular carotenoid is associated with its precise deposition into feathers, and account for organismal coordination in follicular responses across ornaments.

The evolutionary stability of an adaptation can be maintained by interconnectedness of its components or by homeostatic controls that maintain its functionality as a whole. Correspondingly, the transition between adaptations can involve either modifications of individual components or changes in the homeostatic controls that buffered a preceding adaptive state^58^. Here, we distinguished between these scenarios by directly studying the transition between adaptations. We show that variable feather structure induced by uptake of unfamiliar carotenoids during an avian range expansion was consistently converted into precise and stable coadaptations of feather development and carotenoid accommodation as populations persisted. We found that this conversion is underlain by a uniform increase in the sensitivity of feather development to local carotenoid uptake and organism-wide coordination of feather responses. Thus, the mechanism assuring functionality of each adaptive state also likely bridges the transition to new states.

## Methods

### Data collection and sample sizes

We collected 10–15 ornamental feathers (3–5 from each of the three ornamental areas—crown, breast, and rump, Fig. 1 in ref. ^59^) from 1196 free-living adult male house finches. To minimize temporal variation of feather wear and standardize sampling across long-term study populations, we only used feathers sampled during May–June from 2008 to 2018. Captures and sampling were conducted under US Federal Permit (23182) and annual permits for the states of Arizona and Montana. All animal procedures were approved by the University of Arizona Institutional Animal Care and Use Committee (13-423). To ensure sufficient sample sizes for some analyses, we combined populations that shared colonization age and routes (Fig. 3) to nine regions as shown in Supplementary Table 1, which also lists the curated dataset used in all analyses.

Each collected feather was digitized with a modified Epson Perfection 1660 PhotoScanner (Long Beach, CA, USA) at 1000 dpi and also examined under a Nikon Eclipse Ti-E inverted light microscope (Nikon, Tokyo, Japan). Following assessment of feather microstructure, we extracted 19 carotenoid compounds from the pigmented parts of these feathers and obtained concentrations of these carotenoids by HPLC^23^ (µg/g of pigmented feathers, Supplementary Data 1 and 2). Feathers within each ornamental area of each individual were combined for carotenoid extraction and analyses.

### Carotenoid extraction and quantification

Feathers were trimmed, and the weighed pigmented portions were washed in hexane using Whatman GF/A glass filters and finely ground in 3 mL methanol for 10 min at 20 Hz using a Retsch MM301 mixer mill (Newtown, PA), equipped with ZrO grinding jars and balls. Carotenoids were extracted using a 0.2 µm filter (GHP Arcodisc 13 mm Minispike; Pall Life Sciences, East Hills, NY) and the filtrate was dried under vacuum at 40 °C and reconstituted in 150 µL of HPLC mobile phase (methanol:acetonitrile 50:50, v/v).

Carotenoids were quantified by injecting 50 µL of pigment extract into an HPLC System (Shimadzu Corporation, Pleasanton, CA) fitted with an YMC Carotenoid 5.0 µm column (250 × 4.6 mm) and guard column (YMC America, Allentown, PA). Analytes were eluted at a constant flow rate of 1.1 mL/min using isocratic elution with 42:42:16 (v/v/v) methanol:acetonitrile:dichloromethane for the first 11 min, followed by linear gradient up to 42:23:35 (v/v/v) methanol:acetonitrile:dichloromethane through 21 min, isocratic elution at this condition until 30 min when it returned with step function to the initial isocratic condition at which it was held until 40 min. Carotenoids were detected using a Shimadzu SPD-M10AVP photodiode array detector, and data were collected from 200 to 800 nm. Peak areas were integrated at 450 or 470 nm depending on the absorbance maximum (*λ* max) for each compound.

### Carotenoid identification

Fifteen μg/mL stock solutions of pure powdered carotenoid compound dissolved in a mobile phase of 50:50 methanol:acetonitrile were made for 3′-hydroxy-echinenone, 3′-dehydrolutein, α-carotene, adonixanthin, adonirubin, astaxanthin, β-carotene, β-cryptoxanthin, canthaxanthin, echinenone, lutein, retinol, rubixanthin, tocopherols, vitamin D, and zeaxanthin. Five serial dilutions of each carotenoid standard (15, 7.5, 3.75, 1.875, and 0.9375 μg/mL) were run individually through the same HPLC protocol as used for the feather samples above. The serial dilutions enabled us to identify the unique shape of a standard’s peak and determine whether the retention time of a standard shifts in different concentrations. Following the identification of the standard peaks, over 20 unique mixtures of 4–5 known standards of known concentrations were run through the same HPLC protocol as the feather samples to assess how the peaks of different compounds separate when mixed with other compounds, since this is how they are analyzed in the feather samples. As a result of these trials, peaks in feather samples were assigned to compounds based on three key, repeatable features: retention time in the chromatogram, relative retention time to other peaks in the sample, and the shape of the peak.

For fourteen carotenoid compounds, the concentrations of compounds (µg/g) were calculated using calibration curves of these standards (Sigma-Aldrich, St. Louis, MO; Indofine Chemical, Hillsborough, NJ; CaroteNature, Ostermundigen, Switzerland; Santa Cruz Biotechnology, Dallas, TX). For five compounds without available standards, concentrations were derived based on biochemical structural similarity to known standards as follows: the peaks of canary xanthophyll A and canary xanthophyll B were identified based on their proximity to the known peak of 3′-dehydrolutein, 4-oxo-rubixanthin was the peak that appears in the rubixanthin standards when they are exposed to oxygen, the peak for gaziaxanthin was identified based on its proximity to the known peaks of rubixanthin and 4-oxo-rubixanthin, the peak for β-isocryptoxanthin—based on its proximity to the known peak for β-carotene, and the peak for α-doradexanthin—based on its structural similarity to canthaxanthin. We used the serial dilutions to calculate the concentration equation for each standard. The equation was derived from a linear regression of the area of the peaks at the five known concentrations (15, 7.5, 3.75, 1.875, and 0.9375 μg/mL). The concentration of a compound in a feather sample was determined by entering the area of its peak into the equation for its corresponding standard. Supplementary Data 1 and 2 list prevalence of identified carotenoids in feathers (% of individuals), mean and range of their concentration, proportion of the total amount of carotenoids in pigmented feather, and coefficient of variation of that proportion.

### Carotenoid grouping

Carotenoids were grouped into three categories based on their metabolic derivation and connectivity (Supplementary Fig. 1). Dietary carotenoids (external carotenoids deposited into feathers unmodified) are lutein, (3R,3′R)-zeaxanthin, β-cryptoxanthin, α-carotene, gazaniaxanthin, rubixanthin, and β-carotene. Metabolically-derived, nondegenerate carotenoids (those derived from a single dietary compound through 1–2 reactions) are canary xanthophyll A, canary xanthophyll B, β-isocryptoxanthin, echinenone, 4-oxo-rubixanthin, and a-doradexanthin. Metabolically-degenerate carotenoids (those derived from two or more biochemical pathways of similar lengths starting from more than one dietary precursor) are (3S,3′R)-adonixanthin, adonirubin, (3S,3′S) astaxanthin, 3′-dehydrolutein, 3′-hydroxy-echinenone, and canthaxanthin (Supplementary Fig. 1). Although canary xanthophyll B can be produced by pathways starting from two dietary compounds (Supplementary Fig. 1), comparison of the flux of a one-reaction-long path from dietary lutein compared with the flux of two-reactions-long path from dietary zeaxanthin showed that this compound is overwhelmingly produced only by transformation of lutein^60^. We thus categorized canary xanthophyll B as nondegenerate carotenoid for analyses in Figs. 6 and 7.

### Feather differentiation

Differentiation of collected feathers was assessed in two ways. First, using high resolution scans of all feathers, we examined the microstructure of four feathers from each ornament to derive a mean score of barbule loss within each ornamental area of each bird (Supplementary Data 3, see below). Second, we examined one feather from each ornament of *n* = 217 males under a light microscope at ×4, ×6, and ×10 magnifications. For *n* = 651 feather samples in this subset, we assigned a barbule loss score to confirm reliability of the first method, and also measured the proportion of the barb that did not develop barbules relative to total barb length to derive percentage of barbule loss. We then measured barb width at the base, middle and distal tip of barb (Supplementary Data 3). All measures of differentiation were performed on the two most distal, innermost barbs. We assigned a measure of differentiation as follows: 0—no response: full structure, barbules from base to tip (Fig. 1c, d), including feathers with full structures, but partially broken barbules, 1—partial response: partial structure, some barbules developed while others did not separate from barb (Fig. 1e), 2—full response: no barbules present (Fig. 1a, b). All measures of barbule loss were performed by a single observer. The measurement error, assessed in repeated measures of a subsample of 40 crown feathers using one-way ANOVA was less than <5% of the individual identity effect (mean squares 0.06 vs. 1.24).

### Assessment of distribution shape and 50% response threshold

To derive response curve and response threshold values, we needed a technique that would accommodate continuity in response assessments (values 0–2), but also discreteness of these categories. Further, the method needed to accommodate differences in the response distribution—e.g., threshold vs. linear, symmetric around zero vs. accelerating. Thus, we used PROC PROBIT in SAS 9.4, which satisfies these requirements, to calculate the threshold response (our L50 parameter is LD50 probability value in PROC PROBIT), parameter estimates, and the response distribution patterns. The procedure obtains and compares maximum likelihood estimates from linear, probit, logit, ordinal logistic, and accelerating value regression models. Supplementary Data 4 lists calculated threshold response (L50) for the best fit models. Supplementary Fig. 3 outlines the sequence of tests for comprehensive assessment of response threshold and shape.

### Assessment of scaling and deviation from scaling

A change in the shape of feather response in relation to carotenoid concentration can be due to changes in the threshold of concentration that causes a 50% response in a population or changes in the shape of this response (Supplementary Fig. 3). A change in shape can be caused either by a scaling of response—e.g., stretching or compression of the *x*-axis (carotenoid concentration) associated with L50 value (Supplementary Fig. 3c) or a different pattern of response—such as linear or sigmoid (Supplementary Fig. 3d). To identify changes in the response curve that were produced by simple rescaling of population- and ornament-specific responses to carotenoid uptake (e.g., when twice higher concentration is associated with the same percentage of response in one population vs. another), we applied Kolmogorov–Smirnov (K–S) tests to residuals from an AFT regression model, as implemented in PROC PHREG of SAS 9.42. We fitted the regression model to each ornamental area separately, with all study populations combined, but kept identity of populations for regression grouping (Supplementary Fig. 4). The AFT model is particularly appropriate for our analyses because it assumes a parametric form for both the feather response and carotenoid uptake. We then used the K–S test to assess the probability that K–S maximum distance (Supplementary Data 4, double-headed arrow on Fig. 7 inset) between estimated distribution function (EDF) and observed scaling function are obtained by chance. We derived EDFs of feather responses separately for residuals of each population, relative concentration of each carotenoid, and ornament area and then compared these EDFs with observed scaling functions with the K–S framework implemented in PROC NPAR1WAY of SAS 9.42 for two class comparisons. The procedure computes the maximum deviation of the EDFs and outputs values where the maximum deviation occurs, the two-sample K–S statistic *D* and associated probability that *D* is greater than the observed value under the null hypothesis of no difference between the two distributions. Across all ornamental areas, |K–S max distances| ≥ 1.49 were different from 0 under *P* < 0.05 (Supplementary Fig. 4), such that responses with |K–S| < 1.49 come from the same underlying distribution and differ only by rescaling the concentration of carotenoids needed for response, whereas residuals above dashed line in Fig. 7 correspond to responses that differ more than just in sensitivity of response to concentration (Supplementary Fig. 3, all data and tests in Supplementary Data 4).

### Assessment of anticipatory and induced response

To measure organism-wide coordination of feather response, i.e., the indirect effects of carotenoids accumulated in other feathers on feather response in a focal area (e.g., paths c1 → t2, c2 → t3 in Fig. 4), we needed a technique that would allow us to directly compare responses even though they might come from different distributions (e.g., linear vs. threshold; Supplementary Data 4). This ruled out commonly used multiple least-square regression models or structural equation models (e.g., path analysis). We instead used a new procedure for indirect response comparison implemented in logistic regression models of SAS 9.4.

The plots of (1-specificity) by sensitivity form the receiver operating characteristic (ROC) curves (Fig. 8 inset). Diagonals of these plots correspond to an area under the curve (AUC) of 0.5 and represents the area where the fraction of true positives and false negatives are equal and hence the effect is not different from random. The new ROCCONSTRAST procedure in PROC LOGISTICS of SAS 9.42, uses a nonparametric test to compare AUC and associated probabilities, allowing direct comparisons of AUC that come from different distributions (e.g., Supplementary Fig. 5). For each carotenoid compound, we used a focal feather response as a reference point, and compared indirect effects of carotenoids influencing response of feathers in a focal ornament (e.g., c1 → t1, Fig. 4) to the feather response in other ornaments. Values significantly different from 0.5 (and thus nonrandom) are reported in Supplementary Data 4. Figure 8 plots absolute amounts by which these significant values exceed 0.5.

### Reporting summary

Further information on research design is available in the Nature Research Reporting Summary linked to this article.

## Supplementary information

Supplementary Information

Reporting Summary

Description of Additional Supplementary Files

Supplementary Data 1

Supplementary Data 2

Supplementary Data 3

Supplementary Data 4

## Data Availability

All data are available in the paper and the Supplementary Materials.
