# Supplementary material for: Turning induced plasticity into refined adaptations during range expansion
Source: Nat Commun. 2020 Jun 26;11:3254. doi: 10.1038/s41467-020-16938-7 (PMC7320023; doi:10.1038/s41467-020-16938-7)
Supplement: Supplementary file 1 — Supplementary Information [file 41467_2020_16938_MOESM1_ESM.pdf]

Supplementary Information

Turning induced plasticity into refined adaptations during range expansion

Potticary et al.

Table of content:

Supplementary Figure 1. House finch carotenoid synthesis network indicating groups of carotenoids.
Supplementary Figure 2. Timing and location of carotenoid deposition in feather development.
Supplementary Figure 3. Assessment of changes in the pattern of feather response to carotenoid concentration across populations of different ages.
Supplementary Figure 4. Assessment of deviation from scaling.
Supplementary Figure 5. Assessment of sensitivity of feather response in a focal ornamental area to carotenoids exerting response in other ornamented areas.
Supplementary Table 1. Combined dataset and sample sizes for concentrations of 19 carotenoids (Supplementary Data 1, 2) and feather differentiation (Supplementary Table 2) across study populations.
Supplementary Table 2. Prevalence and extent of feather differentiation across study populations.

Supplementary References

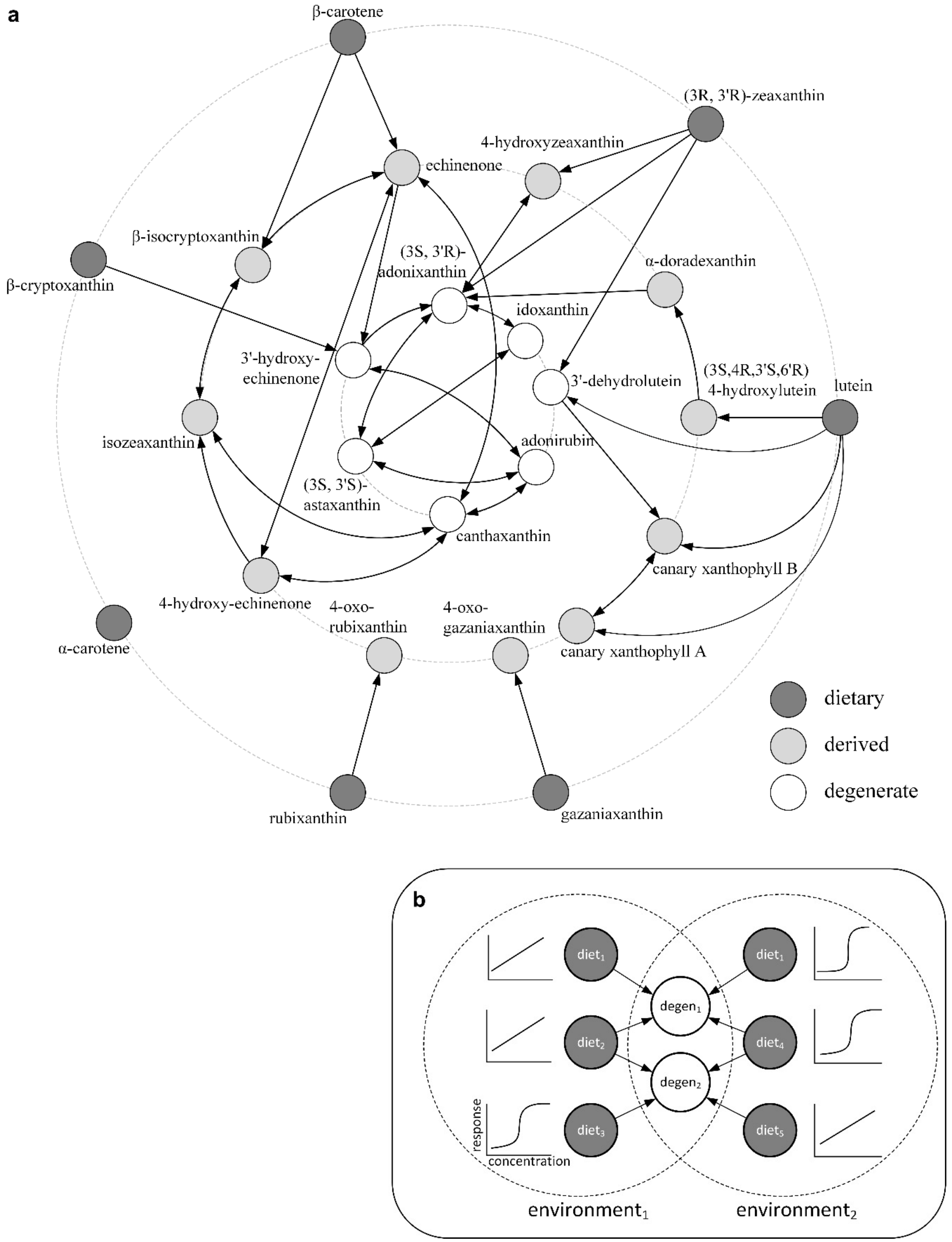

Supplementary Figure 1 a) House finch carotenoid synthesis network indicating groups of dietary (dark grey, outer circle), non-degenerate derived (light grey, middle circle), and degenerate (white, inner circle) carotenoids. Based on ref. [1,2]. **b)** Dietary carotenoids ($diet_n$) vary across two environments, forming variable associations with feather differentiation (inset graphs). Metabolically-derived carotenoids, which are interchangeably produced by different dietary precursors (degenerate carotenoids, $degen_n$), persist in an organism in both environments, and thus allow closer coevolution with feather structure.

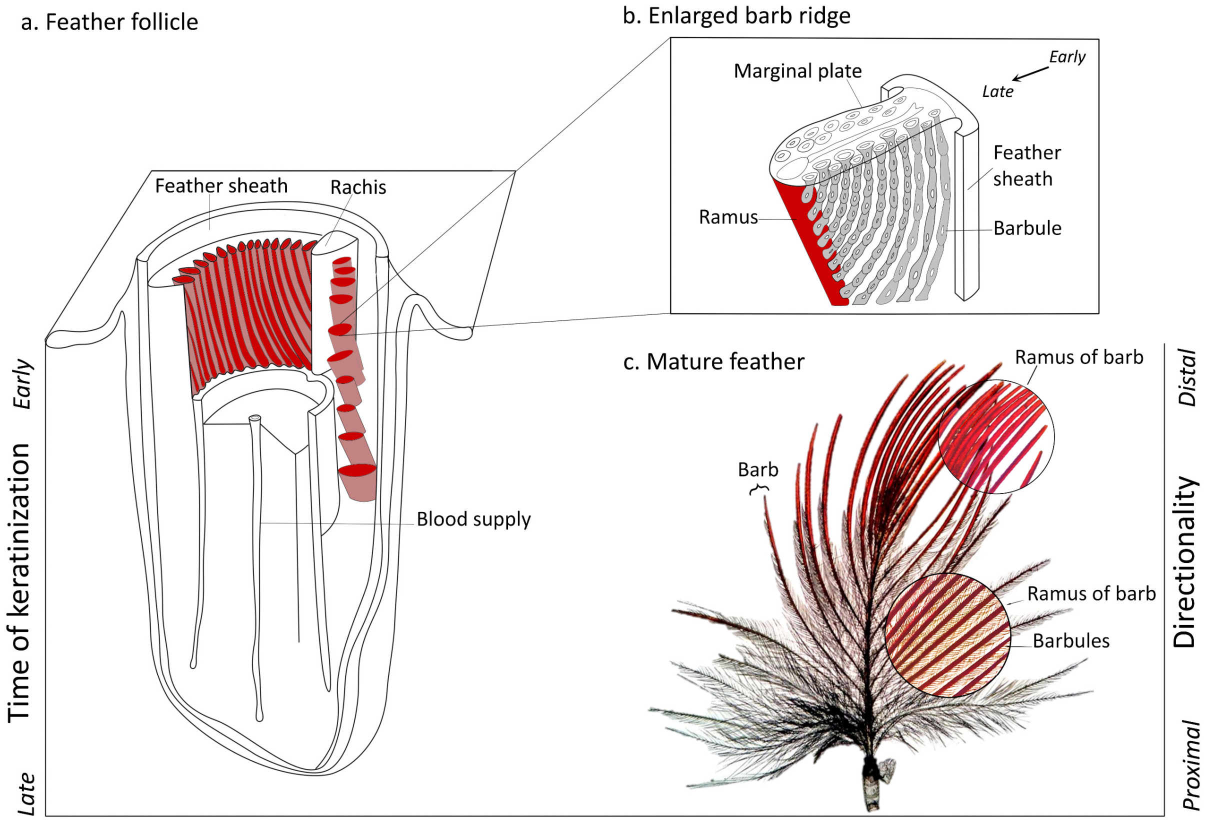

Supplementary Figure 2 Timing and location of carotenoid deposition in feather development. Keratinization occurs when blood and nutrient flow are truncated and final structures are set in keratin. Keratinization proceeds distally-proximally and posteriorly-anteriorly. The proximal end of the feather attaches to the epidermis. Red coloration indicates location of carotenoid deposition in the ramus of barbs. **a)** The feather follicle is a columnar structure, where developing barbs are supplied with nutrients and carotenoids from capillaries in the pulp. Carotenoids diffuse through the pulp epithelium and are taken up by the rami of developing barbs. Barbules are immediately adjacent to the feather sheath, whereas the rami of developing barbs are adjacent to pulp epithelium. **b)** Development of barbules proceeds from outside (feather sheath) in, with the ramus keratinizing last, as it must maintain some flow of nutrients for the proliferation of barbules. Directionality of keratinization is indicated by “Early” to “Late” arrows. **c)** Ornamental crown feather of a house finch. Whole barb is indicated with brackets, inset shows two substructures of barb, including the ramus of barb and branching barbules in between.

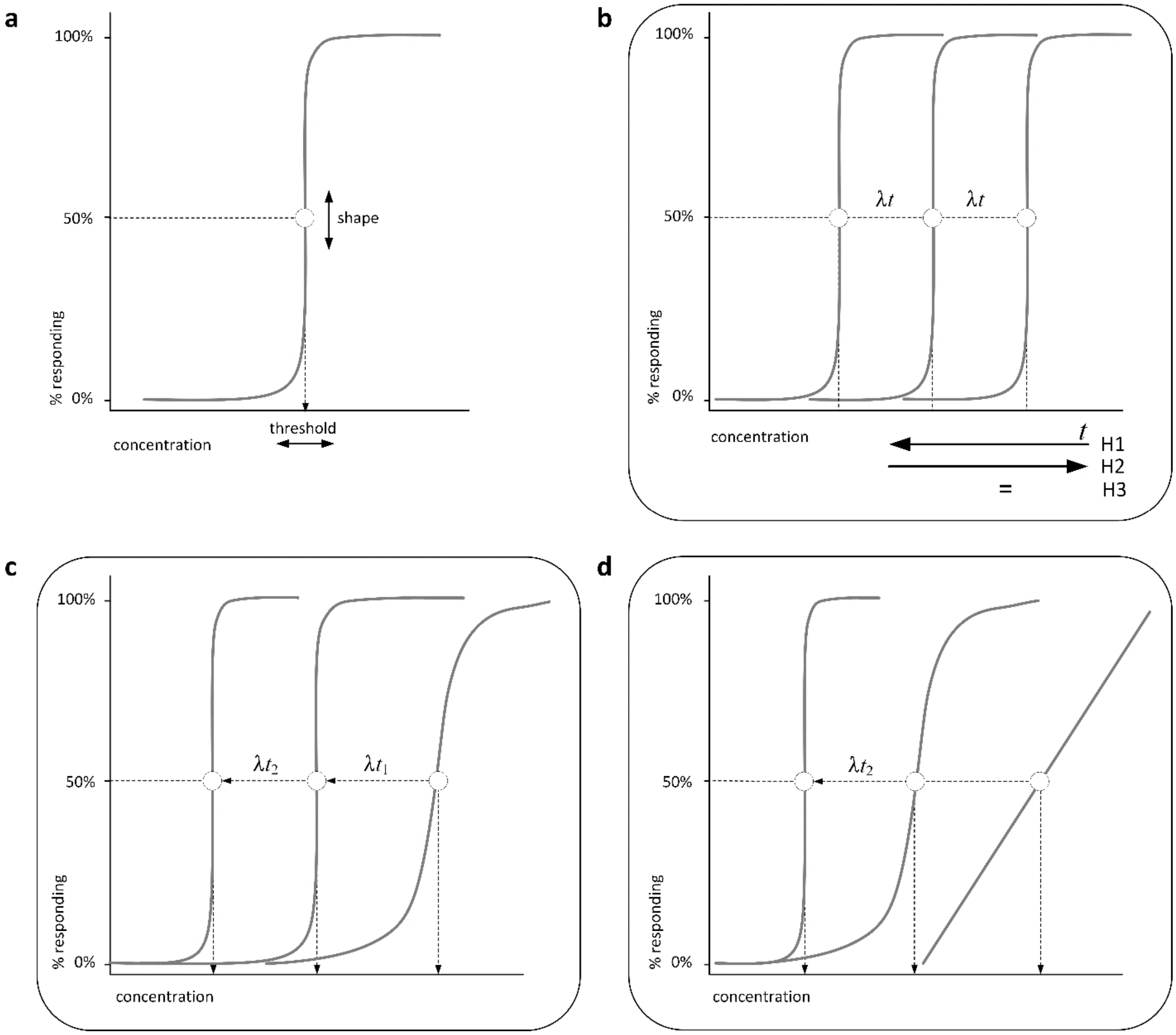

Supplementary Figure 3 Assessment of changes in the pattern of feather response to carotenoid concentration across populations of different ages. a) Relationship between concentration of carotenoid (x-axis) and fraction of a population showing response to this concentration (y-axis) is described by i) the threshold at which 50% of a population shows a response (*L50*) and variation in this threshold (double arrow on x-axis) and ii) the shape of the relationship between carotenoid concentration and population response and variation in this shape (double arrow on y-axis). Here we examine historical changes in both the threshold (Fig. 6) and the shape (Fig. 7) of feather response. **b)** *L50* threshold is predicted to decrease with the time (t) of population persistence under Hypothesis 1 (H1, Fig. 2b), increase with the time of population persistence (H2, Fig. 2c) or remain unchanged over time (H3, Fig. 2d). The shape of this relationship can remain the same as in b) or change either in **c)** scaling, where λ is scaling coefficient (given in Supplementary Data 4) or **d)** in the pattern of response (linear, sigmoid, no relationship) as shown in Supplementary Data 4. Scaling here means that carotenoid concentration associated with *L50* varies by scaling coefficient only – e.g., decrease or increase in L50 concentration. Higher λ here means *lesser* concentration is sufficient to achieve response of 50% of individuals (left-most curve in c), lower λ means *greater* concentration is required to achieve full response of 50% of individuals (right-most curve in c). If response curves differ by scaling coefficient only, their residual from accelerated failure time (AFT) regression will not be different from zero (Fig. 8). If the pattern of the response differs across times of sampling, as shown on **d)**, then ATF residuals will deviate from zero, requiring the analysis appropriate for comparison of different shapes of response (Figs. 6 and 8, Supplementary Data 4). The results of this study are most consistent with **H1** and **c)** for degenerate and other metabolically derived carotenoids, and **d)** for dietary carotenoids.

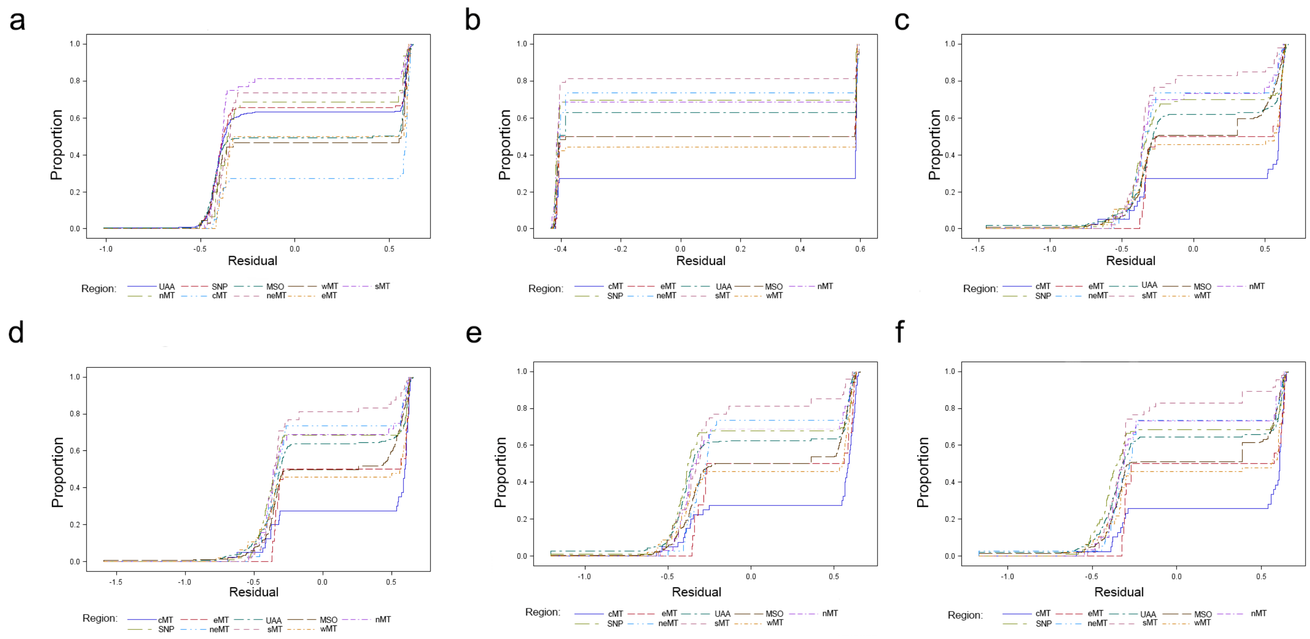

Supplementary Figure 4 Assessment of deviation from scaling. Examples of distributions of residuals from accelerated failure time regression of feather differentiation response to a carotenoid concentration across study populations. The residuals from this regression were used for Kolmogorov-Smirnov tests for deviation from perfect scaling (Fig. 7). Shown are distributions for breast feathers in reaction to concentration of **a)** lutein, **b)** β-carotene, **c)** echinenone, **d)** 3'-hydroxy-echinenone, **e)** adonirubin, and **f)** canthaxanthin.

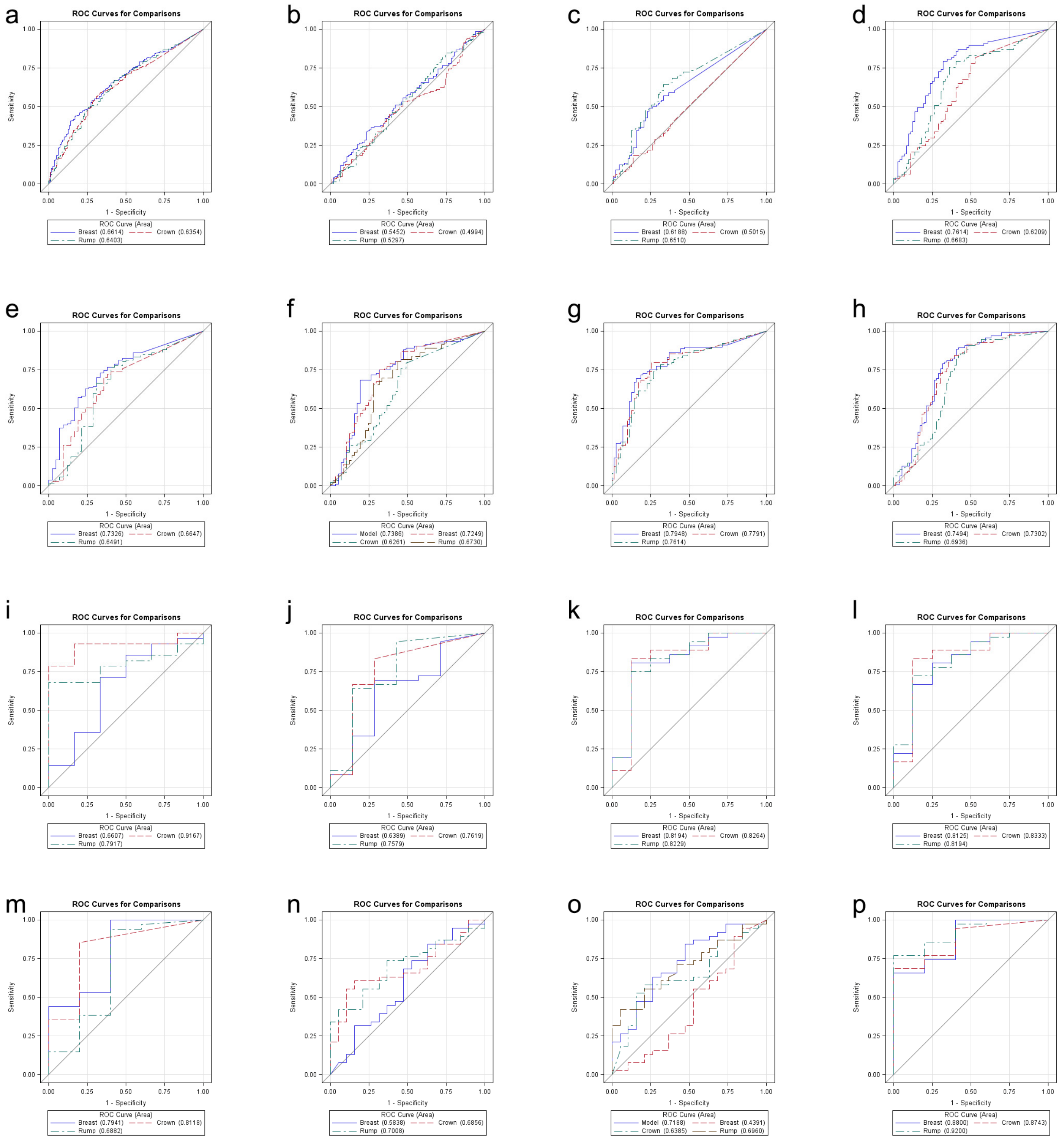

Supplementary Figure 5 Assessment of sensitivity of feather response in a focal ornamental area to carotenoids exerting response in other ornamented areas. Greater area above diagonal indicates greater sensitivity. Areas from different distributions (Supplementary Data 4) can be compared directly (ROCContrast procedure of SAS 9.14) and only areas significantly different from 0.5 are shown in Supplementary Table 5. Shown are examples of **a)** high organismal integration of responses, **b)** lack of integration, **c)** breast feathers and response to α-carotene in other ornaments in MSO population, **d)** breast feathers and (3R,3'R)-zeaxanthin in MSO, **e)** crown feathers and (3R,3'R)-zeaxanthin in MSO, **f)** rump feathers and (3R,3'R)-zeaxanthin in MSO, **g)** breast feathers and (3S,3'S) astaxanthin in MSO, **h)** breast feathers and echinenone in MSO, **i)** breast feathers and echinenone in neMT, **j)** breast feathers and β-isocryptoxanthin in sMT, **k)** breast feathers and echinenone in sMT, **l)** breast feathers and 3'-hydroxy-echinenone in sMT, **m)** breast feathers and (3S,3'S) astaxanthin in sMT, **n)** breast feathers and (3R,3'R)-zeaxanthin in sMT, **o)** rump feathers and (3R,3'R)-zeaxanthin in sMT; **p)** crown feathers and (3R,3'R)-zeaxanthin in wMT population.

Supplementary Table 1. Combined dataset for sampling relative concentrations of 19 carotenoids (Supplementary Data 1, 2) and feather differentiation (Supplementary Table 2) across 43 house finch populations.

Region<br>**abbreviation**	Populations (sampling locations, latitude, ºN, longitude, ºW)	Complete samples*<br>feathers/males
Saguaro National Park<br>**SNP**	Saguaro National Park West (32.2888, 111.1533)<br>Saguaro National Park East (32.2064, 110.6884)	390/129
UArizona Arboretum<br>**UAA**	U Arizona campus/arboretum (32.2319, 110.9501)	981/327
Southern Montana<br>**sMT**	Billings (45.76023, 108.60074; 45.77054, 108.587661)<br>Harlowton (46.43424, 109.8386)<br>Round Up (46.449328, 108.538722)<br>Winnett (47.004347, 108.35399)<br>Livingston (45.6614, 110.5600)<br>Dillon (45.21415, 112.62576; 45.21579, 112.62514; 45.21193, 112.625503)<br>Dell (44.7230, 112.6972)<br>Lima (44.635406, 112.592824)<br>Butte (46.00752, 112.5527; 46.01031, 112.55066)<br>Anaconda (46.13261, 112.97041)<br>Three Forks (45.89345, 111.55875)<br>Helena (46.620669, 112.038039; 46.575485,112.00181; 46.565, 111.978302; 46.611276, 111.920336)<br>White Sulphur Springs (46.55096, 110.90036; 46.54693, 110.90546)	359/123
Missoula area<br>**MSO**	Vigilante MiniStorage (46.8787, 113.9966)<br>Paws Up (46.916957, 113.435576)	990/330
Eastern Montana<br>**eMT**	Broadus (45.44769, 105.4053)<br>Miles City (46.40561, 105.82799; 46.39659, 105.83289)<br>Circle (47.4156, 105.5907)<br>Culberson (48.147585, 104.514955)	96/42
Northeastern Montana<br>**neMT**	Malta (48.352662, 107.880582)<br>Westby (48.8696, 104.0511)<br>Plentywood (48.77368, 104.547787)<br>Glasgow (48.202811, 106.634599)<br>Fort Peck (48.0089, 106.4486)	116/50
Central Montana<br>**cMT**	Choteau (47.81437, 112.19193; 47.81847, 112.18762; 47.810427, 112.192737)<br>Augusta (47.4912, 112.39791)<br>Fairfield (47.615545, 111.98016)<br>Great Falls (47.49839, 111.22444)	131/60
Northern Montana<br>**nMT**	Chester (48.5105, 110.9675)<br>Havre (48.5500, 109.6841)<br>Cut Bank (48.629812, 112.333318)<br>Valier (48.305658, 112.251628)	91/66
Western Montana<br>**wMT**	St. Regis (47.3024, 115.0991)<br>Kalispell (48.21943, 114.33305)<br>Big Fork (48.0633, 114.0726)<br>Superior (47.193045, 114.888817)<br>Plains (47.459956, 114.884694)<br>Polson (47.687447, 114.160314)<br>Hamilton (46.246863, 114.173587; 46.271587, 114.15733; 46.288764, 114.09779)<br>Thompson Falls (47.600191, 115.342068)	184/69

*- feather scores and carotenoid concentration profile for all three ornamental areas

Supplementary Table 2. Prevalence and extent of differentiation of ornamented feathers across house finch populations. Shown are frequencies of full response (individuals with at least one completely non-differentiated feather, %full) and the complete lack of response (all feathers are differentiated, %none), average response (BarbScore) and average percent of undifferentiated feather (PercScore) and its coefficient of variation (CV).

	Breast						Crown						Rump				
Region	%full	%none	BarbScore	PercScore			%full	%none	BarbScore	PercScore			%full	%none	BarbScore	PercScore	
			mean±se	mean±se	CV				mean±sem	mean±se	CV				mean±se	mean±se	CV
SNP	52.4	4.8	1.37±0.06	94±4	27.4		77.0	0.0	1.51±0.03	97±8	23.4		48.2	12.8	1.14±0.05	99±3	24.2
UAA	37.1	14.4	0.79±0.03	87±5	31.0		56.0	10.5	1.04±0.03	81±5	35.8		38.7	11.9	0.82±0.03	80±5	33.3
MSO	42.8	33.1	0.76±0.04	71±13	46.1		60.1	13.2	1.11±0.04	45±11	88.6		51.0	26.2	0.84±0.04	94±6	13.1
wMT	34.7	51.0	0.63±0.11	54±10	72.7		60.0	15.6	1.17±0.11	34±8	101.8		38.0	30.0	0.79±0.1	53±18	89.7
nMT	51.5	27.3	1.03±0.13	39±13	91.2		84.6	11.5	1.46±0.13	30±7	67.8		62.5	31.3	0.96±0.13	58±3	89.3
cMT	17.1	34.1	0.42±006	84±7	37.4		36.6	2.4	0.76±0.09	78±7	43.5		24.4	22.0	0.55±0.07	81±8	44.1
neMT	48.7	20.5	0.93±0.1	50±4	141.4		89.2	0.0	1.62±0.06	34±7	58.5		55.3	7.9	1.0±0.09	70±14	46.1
eMT	45.0	25.0	0.78±0.14	75±10	42.4		65.0	5.0	1.2±0.12	78±14	52.1		50.0	10.0	0.88±0.14	49±10	75.4
sMT	70.8	4.2	1.19±0.08	38±6	78.2		87.2	0.0	1.52±0.07	28±5	102.8		66.7	2.1	1.2±0.08	76±13	52.5

Supplementary References

1 Badyaev, A. V., Potticary, A. L. & Morrison, E. S. Most colorful example of genetic assimilation? Exploring the evolutionary destiny of recurrent phenotypic accommodation. *American Naturalist* **190**, 266-280, doi:10.1086/692327 (2017).

2 Morrison, E. S. & Badyaev, A. V. Beyond topology: Coevolution of structure and flux in metabolic networks. *Journal of Evolutionary Biology* **30**, 1796-1809 (2017).
