# Supplementary material for: Turning induced plasticity into refined adaptations during range expansion
Source: Nat Commun. 2020 Jun 26;11:3254. doi: 10.1038/s41467-020-16938-7 (PMC7320023; doi:10.1038/s41467-020-16938-7)
Supplement: Supplementary file 2 — Reporting Summary [file 41467_2020_16938_MOESM2_ESM.pdf]

nature research

Corresponding author(s): Ahva L. Potticary and Alex Badyaev

Last updated by author(s): 5/17/2020

Reporting Summary

Nature Research wishes to improve the reproducibility of the work that we publish. This form provides structure for consistency and transparency in reporting. For further information on Nature Research policies, see Authors & Referees and the Editorial Policy Checklist .

Statistics

For all statistical analyses, confirm that the following items are present in the figure legend, table legend, main text, or Methods section.

n/a	Confirmed	
☐	☒	The exact sample size (n) for each experimental group/condition, given as a discrete number and unit of measurement
☐	☒	A statement on whether measurements were taken from distinct samples or whether the same sample was measured repeatedly
☐	☒	The statistical test(s) used AND whether they are one- or two-sided<br>*Only common tests should be described solely by name; describe more complex techniques in the Methods section.*
☐	☒	A description of all covariates tested
☐	☒	A description of any assumptions or corrections, such as tests of normality and adjustment for multiple comparisons
☐	☒	A full description of the statistical parameters including central tendency (e.g. means) or other basic estimates (e.g. regression coefficient) AND variation (e.g. standard deviation) or associated estimates of uncertainty (e.g. confidence intervals)
☐	☒	For null hypothesis testing, the test statistic (e.g. F, t, r) with confidence intervals, effect sizes, degrees of freedom and P value noted<br>*Give P values as exact values whenever suitable.*
☒	☐	For Bayesian analysis, information on the choice of priors and Markov chain Monte Carlo settings
☐	☒	For hierarchical and complex designs, identification of the appropriate level for tests and full reporting of outcomes
☒	☐	Estimates of effect sizes (e.g. Cohen's d, Pearson's r), indicating how they were calculated

Our web collection on statistics for biologists contains articles on many of the points above.

Software and code

Policy information about availability of computer code

Data collection	No software was used for data collection
Data analysis	All data analyses were conducted with SAS 9.4

For manuscripts utilizing custom algorithms or software that are central to the research but not yet described in published literature, software must be made available to editors/reviewers. We strongly encourage code deposition in a community repository (e.g. GitHub). See the Nature Research guidelines for submitting code & software for further information.

Data

Policy information about availability of data

All manuscripts must include a data availability statement. This statement should provide the following information, where applicable:

- Accession codes, unique identifiers, or web links for publicly available datasets
- A list of figures that have associated raw data
- A description of any restrictions on data availability

All data are available in the Supplementary Data files

Field-specific reporting

Please select the one below that is the best fit for your research. If you are not sure, read the appropriate sections before making your selection.

☐ Life sciences ☐ Behavioural & social sciences ☒ Ecological, evolutionary & environmental sciences

For a reference copy of the document with all sections, see nature.com/documents/nr-reporting-summary-flat.pdf

Ecological, evolutionary & environmental sciences study design

All studies must disclose on these points even when the disclosure is negative.

Study description	We evaluated structural transformation of feathers associated with uptake of 19 carotenoid compounds across study populations.
Research sample	Male house finches were sampled for 3-5 feathers per three ornamental areas to provide sufficient substrate to extract carotenoids samples per ornament while not affecting the ornament of the bird. All captured males from 43 study populations were sampled and included in analyses (Supplementary Table 1 lists locations and sample sizes).
Sampling strategy	All captured males were sampled. For some analyses (Supplementary Table 1), data from study populations were combined into nine regions that shared the origin, general geographic location, and colonization route.
Data collection	Field data were collected following identical field protocols in all populations, where data were recorded in both a hard copy and a digital copy. All feather structural analyses were conducted following an established protocol by a single observer, where data were uploaded continuously to a digital backup. HPLC and other biochemical analyses were conducted following established laboratory protocols by technicians working under the one supervisor, with data uploaded continuously to a digital backup.
Timing and spatial scale	All males captured between May and June 2008-2018 in 43 study population were sampled for this study
Data exclusions	No data were excluded
Reproducibility	No experiments were conducted in this study. Data on feather differentiation and concentration of 19 carotenoid compounds are included in Supplementary Data files
Randomization	No experiments were conducted in this study.
Blinding	Investigators conducted and completed their component of their work independently of each other and blindly in relation to sample allocation or grouping (Author Contributions detail these components). Datasets were not shared until all the derivations were completed.

Did the study involve field work? ☒ Yes ☐ No

Field work, collection and transport

Field conditions	Data were collected in long-term house finch study populations in Montana and Arizona
Location	Exact locations of all populations and sampling locations are given in Supplementary Table 1.
Access and import/export	All sampling was authorized by US Federal Permit (23182), and annual permits for the stages of Montana and Arizona. Work was approved by IACUC of University of Arizona (13-423).
Disturbance	Data were collected a part of routine field work in long-term study populations and caused minimal disturbance to animals or habitats. Males were captured and released at permanent feeding stations maintained across study sites.

Reporting for specific materials, systems and methods

We require information from authors about some types of materials, experimental systems and methods used in many studies. Here, indicate whether each material, system or method listed is relevant to your study. If you are not sure if a list item applies to your research, read the appropriate section before selecting a response.

Materials & experimental systems

n/a	Involved in the study	
☒	☐	Antibodies
☒	☐	Eukaryotic cell lines
☒	☐	Palaeontology
☐	☒	Animals and other organisms
☒	☐	Human research participants
☒	☐	Clinical data

Methods

n/a	Involved in the study	
☒	☐	ChIP-seq
☒	☐	Flow cytometry
☒	☐	MRI-based neuroimaging

Animals and other organisms

Policy information about studies involving animals; ARRIVE guidelines recommended for reporting animal research

Laboratory animals	This study did not involve laboratory animals.
Wild animals	All birds were captured and measured during routine banding at the established feeding stations using walk-in basket traps and mistnets. All birds were released within 30 min of capture.
Field-collected samples	n/a
Ethics oversight	The study was approved by University of Arizona Animal Care Committee.

Note that full information on the approval of the study protocol must also be provided in the manuscript.
