# Supplementary material for: Turning induced plasticity into refined adaptations during range expansion
Source: Nat Commun. 2020 Jun 26;11:3254. doi: 10.1038/s41467-020-16938-7 (PMC7320023; doi:10.1038/s41467-020-16938-7)
Supplement: Supplementary file 3 — Description of Additional Supplementary Files [file 41467_2020_16938_MOESM3_ESM.pdf]

Description of Additional Supplementary Files

File Name: Supplementary Data 1
Description: Dietary carotenoids. Descriptive statistics of prevalence (Pr, % of individuals), mean and range of concentration (ug/g of pigmented feather), proportion of the total amount of carotenoids in pigmented feather (%tot) and coefficient of variation of that proportion (CV) for dietary carotenoids across study populations of house finches.

File Name: Supplementary Data 2
Description: Metabolically-derived carotenoids. Descriptive statistics of prevalence (Pr, % of individuals), mean and range of concentration (ug/g of pigmented feather), proportion of the total amount of carotenoids in pigmented feather (%tot) and coefficient of variation of that proportion (CV) for across study populations in house finches.* indicates degenerate carotenoids (Supplementary Figure 1), others are non-degenerate derived carotenoids.

File Name: Supplementary Data 3
Description: Raw data on feather differentiation and measurements.

File Name: Supplementary Data 4
Description: Summary of analyses of individual carotenoid compounds across study populations. Listed are response threshold [L50, log10 (concentration)], L50 as a percentage of mean concentration (%M, Fig. 6), patterns of response for breast, crown, and rump ornamental feathers (Ssigmoid, L-linear, -absent), sensitivity of response in focal ornamental feathers to response in other ornamental areas (Br-breast, Cr – crown, Ru- rump, only values significantly different from 0.50 are shown, significant values in excess of 0.50 are plotted in Fig. 8) and absolute values of KolmogorovSmirnov maximum distance (K-S, Fig. 7).
